# Effectiveness of modified cutting and suture technique for endonasal caudal septoplasty in correcting nasal obstruction and preventing nasal tip projection loss

**DOI:** 10.1186/s40463-021-00516-y

**Published:** 2021-06-16

**Authors:** Yu Hosokawa, Takeshi Miyawaki, Taisuke Akutsu, Kazuhiro Omura, Shinya Tsumiyama, Jiro Iimura, Nobuyoshi Otori, Hiromi Kojima

**Affiliations:** 1 Department of Otorhinolaryngology, The Jikei University School of Medicine, 3-19-8, Nishishimbashi, Minato-ku, Tokyo, 105-8471 Japan; 2 Septorhinoplasty Clinic, The Jikei University Hospital, Tokyo, Japan; 3 Department of Plastic Surgery, The Jikei University School of Medicine, Tokyo, Japan; 4 Department of Otorhinolaryngology, Tokyo Dental College Ichikawa General Hospital, Chiba, Japan

**Keywords:** Modified cutting and suture technique, Caudal septoplasty, Endonasal septoplasty, Caudal septal deviation, Nasal obstruction, Nasal tip projection

## Abstract

**Purpose:**

Caudal septoplasty is a difficult procedure. The cutting and suture technique is suitable for caudal septoplasty, but a batten graft is always necessary and bears the risk of nasal tip projection loss. We established a modified cutting and suture technique (MCAST), without using a batten graft, and investigated its effectiveness in correcting nasal obstruction and preventing nasal tip projection loss.

**Methods:**

We retrospectively reviewed the medical records of 22 patients who underwent caudal septoplasty using MCAST. Subjective assessment by Nasal Obstruction Symptom Evaluation (NOSE) score and objective assessment by computed tomography (CT) were performed before and after the surgery. For evaluating nasal tip projection, we asked patients about their awareness of external nasal deformity. Additionally, the nasal tip projection was measured by CT and compared before and after surgery.

**Results:**

The median preoperative NOSE score reduced significantly after MCAST (*P* < 0.001). On CT, the ratio of the area of the convex side to that of the concave side in the anterior portion of the nasal cavity increased significantly after MCAST (*P* < 0.001). All patients were unaware of external nasal deformity. There were no significant differences in the mean preoperative and postoperative nasal tip height and nasolabial angle. The mean supra tip height was significantly greater postoperatively than preoperatively (*P* = 0.02).

**Conclusions:**

The MCAST was useful for correcting nasal obstruction with caudal septal deviation. There was no postoperative loss of nasal tip projection. The MCAST can be suitable for correcting C-shaped caudal deviations without dislocating the caudal septum from the anterior nasal septum.

**Supplementary Information:**

The online version contains supplementary material available at 10.1186/s40463-021-00516-y.

## Introduction

Septoplasty is widely performed for improving nasal obstruction as it is a simple procedure with good postoperative results. However, caudal septal deviations can be difficult to correct and often require reoperation [1]. For caudal septoplasty, it is necessary to expose and treat the caudal strut using endonasal approach, such as hemi-transfixion or open approaches [2]. The procedures for separating from the anterior nasal septum (ANS), suturing the connective tissue around the ANS [2] and nasal septum, and resecting the excess cartilage are complicated. The cutting and suture technique is suitable for caudal septoplasty [3]; however, there are some drawbacks. A batten graft is always necessary, and the nostril on the concave side becomes thick. There is also a risk of losing the nasal tip projection due to misalignment of the overlapping cartilage [4]*.*

We modified the cutting and suture technique so that a batten graft is not required. To overcome the drawbacks of the cutting and suture technique, we changed the cutting site, fixation position, and fixation method of the nasal septal cartilage. In this study, we aimed to investigate the effectiveness of the modified cutting and suture technique (MCAST) in correcting nasal obstruction and preventing nasal tip projection loss.

## Methods

We retrospectively reviewed the medical records of patients who underwent caudal septoplasty using the MCAST performed by a single surgeon between October 2019 and September 2020. Patients’ information, including age, sex, history of septoplasty, history of nasal trauma, and mean follow-up period, were collected. All patients had generalised C-shaped caudal septal deviation without dislocation of the caudal septum from the ANS. Diagnoses were made using endoscopy and computed tomography (CT), and caudal septal deviation was evaluated based on the intranasal and CT findings, which was defined as the deviation of anterior to the inferior turbinate concha and anterior to the piriform aperture [5]. The chief complaint was unilateral nasal obstruction. We investigated the improvement of nasal obstruction and changes in nasal tip projection before and after surgery.

**Fig. 1:**
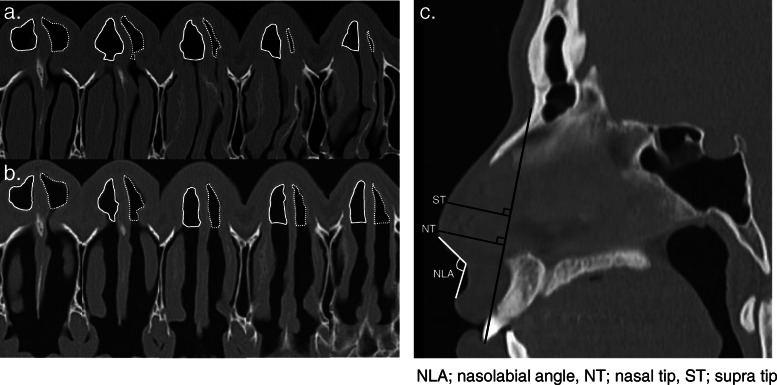
Preoperative (**a**) and postoperative (**b**) areas anterior to the anterior edge of the conchal crest were measured by computed tomography. The dotted lines indicate the area of the convex side, and the solid lines indicate the area of the concave side. **c** For the evaluation of the nasal tip projection, a vertical line was drawn from the line connecting the nasion and maxillary central incisor to the nasal tip and supratip. The distance and nasolabial angle were measured. NLA: nasolabial angle; NT: nasal tip; ST: supra tip

Nasal obstruction status was evaluated subjectively using the Nasal Obstruction Symptom Evaluation (NOSE) score, and objectively using CT, preoperatively and 3-months postoperatively. Changes in nasal obstruction were compared between the preoperative and postoperative NOSE scores and CT. We examined the cross-sectional areas of transverse CT sections. The CT images were acquired at 3-mm thickness in the axial plane. The areas were determined as the regions anterior to the anterior edge of the conchal crest of the maxilla. To measure these areas, images of the nasal cavity were extracted, and the pixels of five images from the anterior nasal spine were calculated (Fig. 1a, b). Finally, we calculated the ratio of the area of the convex side to that of the concave side [6]*.* For the evaluation of nasal tip projection, we asked patients about their awareness of external nasal deformity (yes or no). In addition, the nasal tip projection and nasolabial angle were measured by CT on sagittal view, and compared before and after surgery (Fig. 1a, b). A vertical line was drawn from the line connecting the nasion and maxillary central incisor to the nasal tip and supratip, and the distance was measured. Thus, the nasolabial angle was measured (Fig. 1c). CT analysis was performed using Image J software (National Institutes of Health, Bethesda, MD, USA). CT evaluations of the nasal cavity and external nose were performed by two plastic surgeons (authors T.M and S.T) who did not participate in the surgery. They performed the evaluation while blinded to patient information other than the CT scans.

All continuous variables were treated non-parametrically. Paired continuous variables were compared using the Wilcoxon signed-rank test. Values with two-sided *P* < 0.05 were considered significant. All data were analysed using Stata 15.0 (StataCorp LP, College Station, TX, USA).

### Surgical technique

**Fig. 2:**
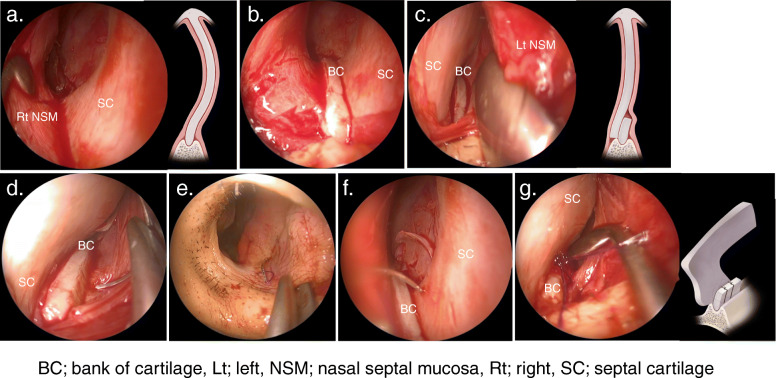
Intraoperative nasal findings. **a**. Elevation of the nasal septal perichondrium flap to correct the posterior aspect of the nasal septum. **b**. The septal cartilage is cut approximately 3 mm above the ANS to create a cartilage bank. **c**. Repositioning the septal cartilage to the medial side. **d**. Penetration of the needle from the middle part of the bank of cartilage to the nasal septal mucosa on the concave side. **e**. Suturing of the concave side of the nasal septal mucosa. **f**. Suturing of the posterior part of the septal cartilage. **g**. The nasal septal cartilage is slid anteriorly and fixed. ANS: anterior nasal septum; BC: bank of cartilage; Lt: left; NSM: nasal septal mucosa; Rt: right; SC: septal cartilage


The nasal septal mucosa was incised using the hemi-transfixion approach. The nasal septal perichondrium flap was elevated to correct the posterior nasal septum, leaving an L-strut of the dorsal and caudal nasal septum of at least 1.5 cm long (Fig. 2a). The septal cartilage was cut approximately 3 mm above the ANS to create a bank of cartilage and trim any excess cartilage (Fig. 2b). The septal cartilage was repositioned medially after pressure release and minimal straightening (Fig. 2c), followed by stitching both bank and septal cartilage to the concave side of the nasal septal mucosa on the ANS (Fig. 2d, e and f). During the first stitch, the septal cartilage was slid anteriorly by suturing the middle of the bank cartilage and the posterior of the septal cartilage (Fig. 2g). The suture was performed with two stitches posterior and anterior to the septal cartilage (see Supplemental Video). The front end of the septal cartilage and connective tissue were sutured to prevent septal cartilage rotation. All MCAST procedures were performed under general anaesthesia and combined with inferior turbinectomy (submucosal resection).

**Additional file 1: Supplementary Material.** MCAST.m4v

## Results

**Fig. 3:**
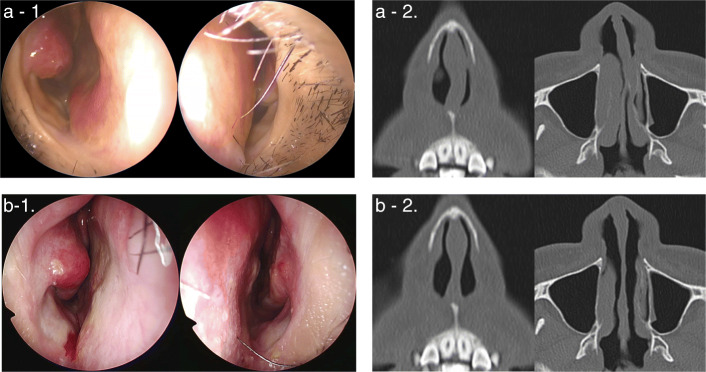
Nasal findings and CT before (**a**-1, 2) and 3 months after (**b**-1, 2) the surgery. **a**-1, 2. Preoperative nasal findings and CT. The left nasal cavity is narrowed by the caudal deviation. **b**- 1, 2. Postoperative nasal findings and CT. MCAST corrected the caudal deviation and equalised the right and left nasal cavities. CT: computed tomography; MCAST: the modified cutting and suture technique

Twenty-two patients (19 males and 3 females) aged 23–67 years (mean ± SD, 36.54 ± 12.56) were evaluated. Two patients had a history of septoplasty, and 6 had a history of nasal trauma. The median follow-up period was 12 months (Table 1). Figure 3 shows the nasal findings and CT before and 3 months after the surgery. The nasal septum, which was strongly curved into the left nasal cavity (Fig. 3-1, 2 a), was corrected and the left nasal cavity was enlarged, allowing the nasal vestibule and cavity to be confirmed (Fig. 3b- 1, 2).

**Table 1:** Patients’ characteristics

Characteristics	*N* = 22
Mean ± SD age, years (range)	36.54 ± 12.56 (23–67)
Sex	
Male (%)	19 (86.36)
Female (%)	3 (13.64)
History of septoplasty (%)	2 (11)
History of nasal trauma (%)	6 (3.66)
Median follow-up period, months (range)	12 (6–16)

*SD* standard deviation

**Fig. 4:**
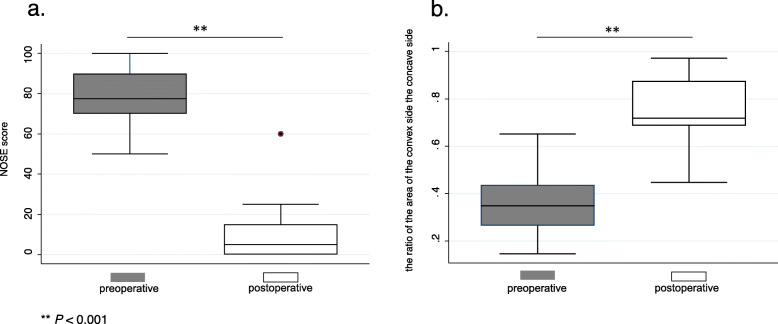
Changes in the mean preoperative and postoperative Nasal Obstruction Symptom Evaluation (NOSE) scores (**a**) and the ratio of the area of the convex side to that of the concave side in the anterior portion of the nasal cavity (**b**). ** *p* < 0.001

The median preoperative and postoperative NOSE scores were 77.5 (interquartile range (IQR), 70–90) and 5 (IQR, 0–15), respectively. The postoperative NOSE score was significantly lower than the preoperative score (*P* < 0.001, Fig. 4a). The median preoperative and postoperative ratios of the area of the convex side to that of the concave side in the anterior portion of the nasal cavity were 0.348 (IQR, 0.265–0.437) and 0.719 (IQR, 0.688–0.876), respectively. This ratio was significantly higher postoperatively than preoperatively (*P* < 0.001, Fig. 4b).

**Fig. 5:**
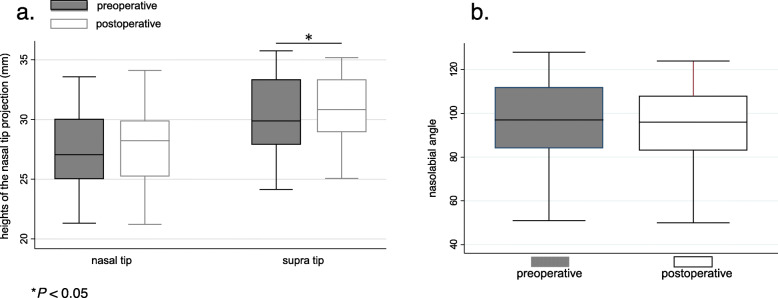
The mean preoperative and postoperative heights of the nasal tip and supra tip (**a**) and nasolabial angle (**b**). * *p* < 0.05

All patients were unaware of external nasal deformity. The mean preoperative and postoperative heights of the nasal tip were 27.24 (IQR, 25.02–30.05) and 27.77 (IQR, 25.22–29.22), respectively. There was no significant change in the height of the nasal tip before and after surgery (*P* = 0.08, Fig. 5a). The mean preoperative and postoperative heights of the supra tip were 30.30 (IQR, 27.89–33.35) and 30.95 (IQR, 28.94–33.37), respectively. The mean postoperative height of the supra tip was significantly higher than the preoperative height (*P* = 0.02, Fig. 5a). The mean preoperative and postoperative nasolabial angles were 96.86 (IQR, 84–112) and 95.81 (IQR, 83–108), respectively. There was no significant change in nasolabial angle before and after surgery (*P* = 0.09, Fig. 5b).

No other complications related to the MCAST were noted.

## Discussion

Correction of the caudal septal deviation is one of the most difficult tasks in septoplasty [7]. Causes of caudal septal deviation can be congenital, iatrogenic, and traumatic [8]. Several studies have described the classification of the shape of the nasal septum [9]. Among nontraumatic septal deformities, caudal deflections, the septal tilt associated with maxillary crests and vomer spurs, and C-shaped and S-shaped deflections are most commonly encountered [2]. This classification is based on surgical approaches and is considered to be clinically useful. The open and endonasal septoplasty techniques are two of the most common surgical approaches used to correct a deviated septum. The open septoplasty is useful for caudal septal deviation [10]. Surgery for heavy deformities such as S-shaped, the multiple fractured and severe malformations require reconstruction with large grafts or extracorporeal septoplasty. Open septoplasty is suitable for these cases. For simple caudal deviation such as C- shaped, both are considered [9]. However, fibrous attachment of the lower lateral cartilage to the septal cartilage and the intercrural ligament is important for nasal tip support [8]. Instability or breakage of the keystones leads to saddle nose deformity [11]. Therefore, important structures such as the lower lateral cartilage and keystone area should be preserved as much as possible. In addition, open septoplasty requires an external incision, which is disinclined to some patients [6]. Thus, a simpler approach that does not entail an external incision would be beneficial for such patients [12]. Therefore, septoplasty should be performed with an endonasal approach whenever technically feasible [2]. In recent years, various endonasal techniques for caudal septoplasty have been reported. The indications of endonasal septoplasty for mild to moderate caudal deviation may expand in the future.

The basis of septoplasty is the adjustment of the convex component of the nasal septum. If the caudal deviation is caused by excess cartilage, the length of the cartilage needs to be shortened. The swinging door technique is widely used for caudal septoplasty [2, 9]. This technique involves separating the septal cartilage from the ANS and adjusting the length of the excess cartilage, after which the ANS and septal cartilage are sutured and fixed. It is a reasonably safe and effective technique, but there is a risk that the septal cartilage might slip from the ANS [4]. In MCAST, the cartilage bank created by cutting the septal cartilage is connected to the ANS. The cartilage bank acts as a stopper for the septal cartilage and therefore is less likely to slip. In addition, by suturing the mucosa and cartilage bank, the nasal septal cartilage is fixed from the left and right sides. Suture fixation is simple because the mucosa is sutured to the cartilage bank instead of the connective tissue.

The cutting and suturing technique is a very good surgical method [3], but its disadvantages are the risk of postoperative external nasal deformity and the fact that a graft is always required [4].. In this study, all patients were unaware of external nose deformity and CT evaluation also showed no loss of tip projection. Rather, the height of the supratip tended to be higher (Fig. 5). In MCAST, the septal cartilage is well preserved. It is thought to maintain the strength in the dorsal-ventral direction. Furthermore, by fixing the nasal septal cartilage anteriorly, the anterior septal angle is slightly rotated dorsally. We considered these are the two reasons that the height of the nasal tip could be maintained without the batten graft. In addition, all patients in this study had C-shaped caudal deviation. Repositioning the caudal septum over the ANS and excising the inferior portion of the caudal strut may be necessary when excessive caudal septum length contributes to the deviation [13]. The length of the caudal septum cartilage in the C-shaped caudal deviation is excessive. Therefore, we consider that cutting 3 mm of the nasal septal cartilage on the inferior side of the C-shaped caudal septum does not cause a loss of tip projection. However, in the case of caudal deviation with dislocation of the caudal septum from the ANS such as the tilt type, the length of the nasal septal cartilage may be normal. In such cases, we considered that cutting downward may lose the tip projection. The swinging door technique or the doorstop technique may be effective in such cases [9]. Thus, MCAST may be suitable for correcting C-shaped caudal deviations without dislocation of the caudal septum from the ANS. However, if the cartilage is cut excessively, there is a risk of pollybeak deformity and other problems that require attention even in the C-shaped caudal septum. Patient education about complications as well as the expected benefits of surgery is important to improve patient satisfaction after surgery [14].

In this study, subjective assessment (NOSE score) revealed a significant improvement (*p* < 0.001). On CT, the ratio of the area of the convex side to that of the concave side in the anterior portion of the nasal cavity was also significantly improved after surgery (*p* < 0.001). There were no complications in this study.

MCAST cannot correct the dorsal deviation. We considered the limit of correction to be the lower three-quarters of the caudal strut. The nasal obstruction has a strong association with nasal airflow and cooling of the nasal mucosa [15]. Casey et al. analysed the correlation between nasal airflow and nasal obstruction in the superior, middle, and inferior areas of the nasal cavity and reported that there was a strong correlation in the middle area [16]. In addition, the nasal vestibule has a high distribution of thermoreceptors [17] and is sensitive to mechanical stimulations [18]. Even if dorsal deviation cannot be corrected, caudal septoplasty improves anterior nasal airflow. Our study results may be attributed to the fact that the correction of the lower three-quarters of the nasal septum increased anterior nasal airflow and improved nasal cooling.

Our study has limitations. The acoustic rhinometry could not be performed as an objective evaluation of nasal obstruction. Since the spread of COVID-19 infection occurred during the study, rhinometry has been banned in our hospital because it is an examination that involves the generation of aerosols. In this study, we did not use patient-reported outcome measures (PROMs) that contain cosmetic aspects, such as the Standardized Cosmesis and Health Nasal Outcomes Survey (SCHNOS) [19]. The use of PROMs is recommended for surgeries that may change the external nasal morphology as well as improve function, such as septoplasty and rhinoplasty [20], and should be used in future studies. Additionally, the number of patients was small, and the follow-up period was short. In the future, it will be necessary to observe the risk of external nasal deformity and recurrence of nasal obstruction in a larger number of patients over a longer term.

## Conclusion

MCAST was useful for correcting nasal obstruction with caudal septal deviation. There was no postoperative loss of nasal tip projection. We believe that this technique may be a new option for caudal septoplasty.

Overall, MCAST can be suitable for correcting C-shaped caudal deviations without dislocation of the caudal septum from the ANS.

## Data Availability

The datasets during and/or analysed during the current study available from the corresponding author on reasonable request.
